# Gaze-Dependent Topography in Human Posterior Parietal Cortex

**DOI:** 10.1093/cercor/bht344

**Published:** 2013-12-18

**Authors:** Jason D. Connolly, Quoc C. Vuong, Alexander Thiele

**Affiliations:** 1 Faculty of Medical Sciences, Institute of Neuroscience, Newcastle University, Newcastle upon Tyne NE2 4HH, UK; 2 Current address: Wolfson Research Institute, University of Durham, Thornaby TS17 6BH, UK; 3 Current address: Department of Psychology, Durham University Science Site, Durham DH1 3LE, UK

**Keywords:** functional magnetic resonance imaging, head-centered, posterior parietal cortex, spatial coordinate frames, topographic mapping

## Abstract

The brain must convert retinal coordinates into those required for directing an effector. One prominent theory holds that, through a combination of visual and motor/proprioceptive information, head-/body-centered representations are computed within the posterior parietal cortex (PPC). An alternative theory, supported by recent visual and saccade functional magnetic resonance imaging (fMRI) topographic mapping studies, suggests that PPC neurons provide a retinal/eye-centered coordinate system, in which the coding of a visual stimulus location and/or intended saccade endpoints should remain unaffected by changes in gaze position. To distinguish between a retinal/eye-centered and a head-/body-centered coordinate system, we measured how gaze direction affected the representation of visual space in the parietal cortex using fMRI. Subjects performed memory-guided saccades from a central starting point to locations “around the clock.” Starting points varied between left, central, and right gaze relative to the head-/body midline. We found that memory-guided saccadotopic maps throughout the PPC showed spatial reorganization with very subtle changes in starting gaze position, despite constant retinal input and eye movement metrics. Such a systematic shift is inconsistent with models arguing for a retinal/eye-centered coordinate system in the PPC, but it is consistent with head-/body-centered coordinate representations.

## Introduction

We take for granted the complex series of neural computations necessary to convert retinal locations into distances relative to the reaching limb. A brain region well suited to perform these operations in human and nonhuman primates is the posterior parietal cortex (PPC). Its position between visual-input (occipital) and motor-output (M1, frontal eye field) areas puts the PPC in a prime locus to integrate visual and motor information. PPC has a topographic representation of visual space much like early visual cortical areas, and it shows a topographic representation related to the endpoints of intended and then executed saccadic eye movements, a so-called saccadotopic map (Sereno et al. 2001). In a saccadotopic map, adjacent saccade endpoints are represented by spatially adjacent locations within each map. That is, adjacent cortical locations in the PPC represent adjacent regions of visual or saccade target space (Sereno et al. 2001; Schluppeck et al. 2005; Konen and Kastner 2008). The topographic representations in the PPC can be influenced by motor input (Mountcastle et al. 1975; Robinson et al. 1978; Colby and Goldberg 1999; Andersen and Buneo 2002). One highly influential theory argues that motor input helps the PPC in the nonhuman primate (and possibly in the human) to encode visual information within an eye-centered coordinate system or reference frame (Batista et al. 1999; Cohen and Andersen 2000, 2002), whereby the position of the eye in the orbit modulates the visual and saccade-related firing of single neurons through corollary discharge (Wurtz 2008) or input from proprioception (Dijkerman and de Haan 2007). Despite substantial support for an eye-centered reference frame in individual neurons of nonhuman primate PPC (Zipser and Andersen 1988; Andersen et al. 1997, 1998; Batista et al. 1999; Buneo et al. 2002, 2008; Cohen and Andersen 2002), it has been argued that PPC neurons employ a hybrid reference frame consisting of a mixture of eye-centered and head-centered neurons (Duhamel et al. 1997; Mullette-Gillman et al. 2005, 2009). The existence of a higher-order coordinate system relative to the head or body beyond the retina or eye, and especially of hybrid coding in the PPC of the human, remains contested. Some functional magnetic resonance imaging (fMRI) studies report data more compatible with a retino-centric coordinate representation (Sereno et al. 2001; Schluppeck et al. 2005; Fernandez-Ruiz et al. 2007; Konen and Kastner 2008; Van Pelt et al. 2010), while others report eye-centered coding (DeSouza et al. 2000; Medendorp et al. 2003). However, there are also recent findings of head- or “face”-centered coding within subregions of the PPC (Sereno and Huang 2006; Pertzov et al. 2011).

To investigate possible coding schemes across the PPC, we exploited the topographic representation of saccade endpoints by having participants make saccades from different starting positions. This allowed us to determine whether and, if so, where higher-order topographic representations such as a head-/body-centered coordinate system exist in the human PPC. If saccadotopic maps are selectively shifted or distorted when retinal stimulation and saccade parameters are held constant, but gaze position is manipulated, this would argue against a retinal/eye-centered coordinate system in the PPC. Using a memory-guided saccade paradigm (Sereno et al. 2001) with different starting gaze positions, we find that topographic maps in lateral/inferior and areas medial to the intraparietal sulcus (IPS) show a global reorganization of saccadotopic space representation across both the inferior and superior parietal lobules of the PPC when gaze is directed to the left or right relative to central gaze. This finding argues against a purely retinal/eye-centered representation and suggests that a substantial proportion of parietal neurons encode visual space in higher-order reference frames, whereby visual space is represented relative to the head or body.

## Materials and Methods

### Subjects

In the present study, we tested 4 male subjects, who had no history of neurological defects. All subjects provided written consent. The study was approved by the Newcastle University Faculty of Medical Sciences Ethics Committee.

### Visual Stimuli and Task

**Figure 1.**
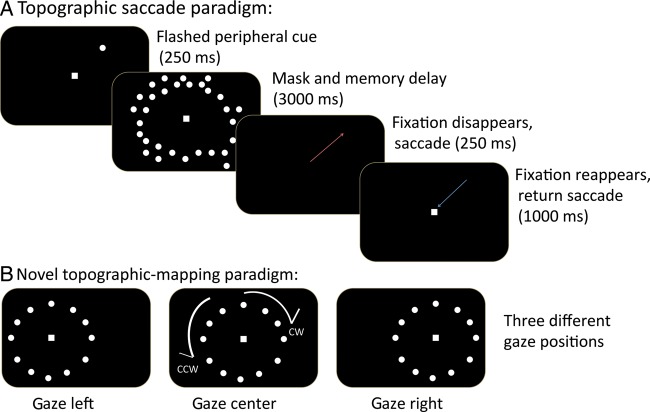
Memory-guided saccade task. (*A*) A standard central gaze topographic saccade paradigm consisted of a peripheral target followed by a memory interval, where a ring of random distracters was presented. Following fixation offset, the subject made a saccade to the remembered location and then returned the eye immediately to central fixation. (*B*) Subjects made such saccades in a sequence to target “around the clock,” either clockwise (CW) or counter-clockwise (CCW). In the LG/RG conditions, subjects made saccades to locations “around the clock” that were shifted 4° either to the left or to the right of the center position.

Each subject performed a memory-guided saccade task (Sereno et al. 2001; Schluppeck et al. 2005; Konen and Kastner 2008) at gaze-center (CG: 0°; center of the screen), gaze-left (LG: −4.1° horizontal shift), and gaze-right (RG: +4.1° horizontal shift) positions (Fig. 1). The positions were run on separate days for 4 of the subjects, and the order was randomly determined for each subject. For each gaze position, a target (a 0.22° high contrast dot) appeared consecutively at 12 locations on an invisible circle centered on the starting gaze position (i.e., the fixation spot). The circle had a radius of 7.7° of visual angle. Like a clock face, each location was separated by 30° starting from the top of the circle (0° or 12 o'clock location; Fig. 1). On each trial, a fixation square appeared at the gaze position for 1 s. A target then appeared for 250 ms, followed by a 3000-ms mask of 100 distracter dots (0.22° high contrast dots). The distracter dots were randomly distributed within an annulus, which had an inner radius of 5.0° and an outer radius of 10.4° from gaze position. When the mask disappeared, subjects made a saccade to the remembered target location and back to fixation (within 250 ms). A complete cycle consisted of a saccade to each of the 12 target locations “around the clock.” Subjects performed 5 cycles per run. Thus, the stimulus periodicity was 5 cycles per run. The total duration of a single run was 270 s (4.5 s per trial × 12 trials × 5 cycles).

Within a session, we alternated scans in which the target advanced in either a clockwise (12 o'clock, 1 o'clock, and so on) or counter-clockwise (11 o'clock, 10 o'clock, and so on) direction. Each subject completed 9–10 runs per scanning session, and 1–2 sessions per starting gaze position were acquired for each subject. Subjects, therefore, performed 540–1200 saccades to remembered locations for each gaze position (CG, LG, and RG). In addition, 1 of the 4 subjects performed a single session in which all gaze positions were tested (30 runs with 1800 saccades). This was done to address any potential confounds due to the gaze conditions being run across separate sessions. We used a Canon XEED LCD projector to project the visual stimuli onto a screen at the foot-end of the scanner. Subjects viewed the screen through an angled mirror attached to the head coil about 10 cm above the subjects' eye. Stimulus presentation was controlled by Psychtoolbox (www.psychtoolbox.org).

### Anatomical Imaging, Registration and Inflated Maps

All MRI data were acquired using a Philips 8-channel receive-only SENSE head coil on a 3-T scanner (Philips Intera Achieva) at the Newcastle Magnetic Resonance Centre. Subjects' heads were stabilized by the use of foam padding behind the back of the head and additional padding between either sides of the forehead and the top of the head coil. A high-resolution, *T*_1_-weighted anatomical volume was acquired in the coronal plane at the beginning of the first session—and the single session for the complete within session dataset—for each subject using a T1TFE pulse sequence (1 mm^3^ voxels). For all sessions, we also acquired a low-resolution, anatomical *T*_1_-weighted volume in the same coronal slice orientation as the functional volumes. An image registration algorithm was used to align our inplane anatomical images from other sessions with the *T*_1_-weighted image (Jenkinson et al. 2002). In a subsequent step, the *T*_2_*-weighted functional dataset was then aligned to the inplane. In this fashion, we were able to average together our clockwise and counter-clockwise scans for a particular starting gaze location across scanning sessions.

The mrTools software (http://gru.brain.riken.jp/doku.php) was used to register the functional data across scanning sessions. We used a combination of FreeSurfer (http://surfer.nmr.mgh.harvard.edu/fswiki) and Matlab-based software from the Heeger Lab (New York University) (http://www.cns.nyu.edu/heegerlab/) to convert the *T*_1_-weighted and segmented Freesurfer image into SurfRelax (http://www.pc.rhul.ac.uk/staff/J.Larsson/software.html). These images were imported into mrTools, which was used to restrict the functional data analyses to gray matter voxels. We then inflated the cortical surface and computed and displayed the multicolored phase maps.

### Functional Imaging

For each functional scan, a *T*_2_*-weighted, echo-planar image pulse sequence was acquired [time repetition: 1500 ms, time echo: 30 ms, flip angle: 75°, 30 slices, 3 mm^3^ voxels, field of view (FOV): 192 mm]. The scans were coronal slices that covered most of the occipital cortex and extended anteriorly to the back of the frontal lobe. Four dummy scans were collected prior to the onset of each functional scan run to eliminate the transient effects of magnetic saturation.

### Preprocessing

We high-pass filtered the time series with a 100-s cutoff at each voxel to remove the slow drift related to fMRI. Data from clockwise and counter-clockwise runs for a given gaze position and within a given session were combined as follows. First, the time series at each voxel was temporally shifted by 3 s to compensate for the hemodynamic lag. We then time-reversed the counter-clockwise runs. Finally, we averaged all runs (clockwise runs + time-shifted and time-reversed counter-clockwise runs) to compute the mean time series for each voxel. Therefore, the mean time series at each voxel represents visual space in a clockwise manner. For our coherence analysis, we computed the percent signal change time series for each voxel by dividing the intensity of each voxel (in arbitrary units) by its mean intensity using mrTools.

### Coherence Analysis (Topographic Mapping)

The percent signal change time series were analyzed by fitting a sinusoid with the same 54 s periodicity as the stimulus cycle to the time series at each voxel. We then computed the coherence between the best-fitting sinusoid and the time series, and the phase of this best-fitting sinusoid. The coherence measures the fit between the reference waveform and the data (from 0 to 1), whereas the phase indicates the temporal delay between the 2 signals that yields the maximum magnitude (from –*π* to *π*). The phase, therefore, corresponds to the direction of the saccade vector relative to the beginning of the stimulus cycle (i.e., 12 o' clock vector). For instance, a 90° phase represents the 3 o'clock saccade vector. The phase at each voxel is then color-coded to visualize the systematic progression of polar angle representation in the PPC, and phase values were equally binned such that the preferred overall phase could be visualized by means of a rose histogram (refer to Figs 4 and 5).

**Figure 2.**
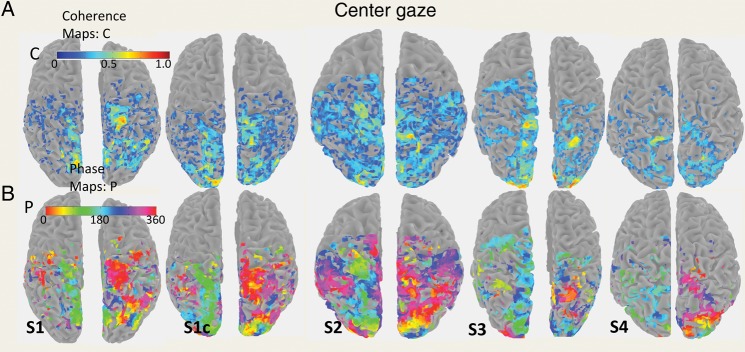
Coherence and phase maps for the gaze-center condition for each subject. The maps show the coherence obtained with certain saccade directions for the 4 across-session subjects +1 within the session, that is, control session (S1c). Left (LH) and right (RH) hemispheres are represented by neighboring plots, with the midline at the center of the LH/RH plots. All maps are thresholded at a coherence value of 0.15. There was significant activity associated with specific saccade directions throughout the medial PPC mantle (e.g., right hemisphere, S1, coherence >0.7, *P* < 0.001), and this was found in every subject. Significant pixels were distributed along the anterior extent of the IPS (green to red pixels) in the LH and violet/blue pixels in the RH. Color bar (C) inset indicates coherence values (0–1.0). Second color bar inset (P) indicates that phases of 0–360°, 0°, and 360° represent upward saccades relative to center, 180° represents downward saccades, 90° represents rightward saccades, and 270° represents leftward saccades.

Visualizations were based on segmenting gray and white matter in the *T*_1_-weighted scans. Activation (coherence) maps (Fig. 2) were confined to the gray matter and were well visualized on the surfaces. We used both anatomical boundaries and boundaries based on previous memory-guided saccade phase-encoded data (Sereno et al. 2001; Schluppeck et al. 2005; Konen and Kastner 2008) to first define the PPC region of interest (ROI). This PPC ROI included voxels anterior to the parieto-occipital sulcus, medial to the IPS, and posterior to the postcentral sulcus. On the inflated surface, this region roughly represented a rectangle. Secondly, we defined PPC subregions IPS1 through IPS5 using an “equidistant procedure,” in which the PPC ROI was further subdivided into 5 separate zones of approximately equal size that encompassed the most posterior to the most anterior portion of the PPC. We also discovered 2 additional regions in the inferior PPC that changed phase preference for left gaze when compared with right gaze. These subregions were labeled IPS6 and IPS7.

### Statistics

The statistical significance of coherence values can be determined by transforming the coherence value (treated as a correlation coefficient) into a *t*-value and then computing the significance with a 2-tailed *t*-test against the null hypothesis *c* = 0 with degrees of freedom = number of trials − 2. (The coherence can be Fisher transformed to normalize the data, but this does not change the significance test much.)

As noted by Schluppeck et al. (2005), there are several assumptions regarding the independence of the time series at each voxel that violate some of the assumptions of the *t*-test. Therefore, the *c*-values (or corresponding *p*) were used only for an initial thresholding of the data to visualize the phase maps. We performed additional analyses to quantify the lateralization (or relative lack thereof) in the case of the gaze deviated maps and the visual field orientations in our anatomically defined subregions of the PPC. Because we were interested in the distribution of phases in our defined ROI, we used a circular statistics package to analyze the data (Berens 2009) (http://www.mathworks.com/matlabcentral/fileexchange/10676).

### Direct Statistical Comparison Between Left and Right Gaze ROIs

For each of the subregions within the PPC (IPS1–IPS7), a circular statistical comparison between the mean phases for the 2 shifted gaze conditions was made using the Watson-Williams test for equal means (Berens 2009). The null hypothesis (H0) was that there would be no difference between the circular distribution of phase values for LG when compared with RG within a particular hemisphere and subregion (Table 1). We further plotted the circular distributions for IPS1 through IPS7 as rose histograms for individual subjects and cumulatively across subjects in order to visualize the effect of gaze shift on topographic representations in the human PPC.

**Table 1:** Results from the Watson-William tests for circular data for the right and left hemispheres

Subregion	Subject	Right hemisphere			Left hemisphere		
		df	*F*-value	*P*-value	df	*F*-value	*P*-value
IPS1	1	1.76	0.10	n.s.	1.28	0.07	n.s.
	2	1.38	1.77	n.s.	1.32	0.03	n.s.
	3	1.46	6.12	<0.05	1.20	1.28	n.s.
	4	1.16	0.09	n.s.	1.30	0.01	n.s.
IPS2	1	1.62	3.54	n.s.	1.20	0.56	n.s.
	2	1.52	9.96	<0.05	1.90	0.59	n.s.
	3	1.58	13.07	<0.01	1.30	1.38	n.s.
	4	1.52	21.35	<0.01	1.30	7.29	<0.01
IPS3	1	1.20	3.64	n.s.	1.32	7.92	<0.01
	2	1.30	0.03	n.s.	1.14	4.98	<0.05
	3	1.28	8.60	<0.01	1.20	0.14	n.s.
	4	1.16	7.48	<0.05	1.32	21.38	<0.01
IPS4	1	1.20	35.51	<0.01	1.42	11.01	<0.05
	2	1.24	2.21	n.s.	1.16	0.15	n.s.
	3	1.26	0.38	n.s.	1.22	0.03	n.s.
	4	1.34	0.57	n.s.	1.22	7.21	<0.05
IPS5	1	1.30	4.07	0.05	1.44	12.00	<0.01
	2	1.16	0.78	n.s.	1.64	59.62	<0.01
	3	1.16	0.25	n.s.	1.28	15.43	<0.01
	4	1.72	12.72	<0.01	1.18	2.17	n.s.
IPS6	1				1.168	220.02	<0.001
	2				1.150	20.96	<0.001
	3				1.88	12.13	<0.001
	4				1.58	22.45	<0.001
IPS7	1	1.94	31.28	<0.005	1.122	114.89	<0.005
	2	1.70	9.47	<0.005	1.174	39.83	<0.005
	3	1.70	8.94	<0.005	1.78	14.68	<0.005
	4	1.32	9.28	<0.005	1.194	6.90	<0.01

Note: The comparisons are listed in the left column. The significance of the effect is indicated by the *P*-value column (uncorrected for multiple comparisons). df: degrees of freedom. Subject 1 represents S1c (or control session).

### Eye Tracking

Eye movements were monitored using a long-throw optics near-infrared scanner compatible eye tracker (Applied Science Laboratories, Bedford, MA, USA). This eye tracker uses bright pupil technology and tracked saccades at 120 Hz. The eye traces were used to verify that the subject was fixating at the proper locations for each of the different “starting gaze” positions. In addition, we further conducted eye tracking on 4 additional subjects off-line using an EyeLink II system (SR Research Ltd, Kanata, ON, Canada) to examine eye movement metrics across the 3 different starting gaze conditions.

## Results

To examine whether the PPC represents saccadic endpoints in retinal/eye-centered or higher-order head-/body-centered coordinate systems, we obtained topographic maps in the PPC using the memory-guided saccade task (Sereno et al. 2001; Schluppeck et al. 2005; Konen and Kastner 2008). Here, subjects make saccades from a central gaze position (where the eyes are in the center of the orbits and aligned with head and body midline) to consecutive peripheral targets “around the clock.” In contrast to earlier studies employing the same task, we collected multiple maps for each subject as a function of starting gaze position.

For the gaze-center condition, we replicated the topography related to saccade direction that has been previously demonstrated in the human PPC (Sereno et al. 2001; Schluppeck et al. 2005; Konen and Kastner 2008), although our maps did not exhibit the very gradual progression of phase values of these previous studies. For this reason, we used an “equidistant parsing” of IPS1–IPS5 (see Materials and Methods). The coherence maps thresholded at *c* > 0.15 obtained for the gaze-center condition are shown in Figure 2*A* (top panel). There was a continuous high-coherence band medial to the IPS in both hemispheres of all 5 datasets (4 subjects + 1 control session, or S1c). In each activated subregion (IPS1–IPS7), there were voxels with coherence values above *c* > 0.2 (*P* < 0.035, uncorrected), and in the majority of these regions, there were voxels with *c* > 0.7 (*P* < 0.001). Figure 2*B* (bottom panel) shows the center-gaze phase map for individual subjects with the corresponding coherence maps thresholded at *c* > 0.15. The phase values in the antero-medial PPC demonstrates that high-coherence values (those with values of *c* approaching >0.7) in the left and right hemispheres were shifted by a phase difference close to *π* (Fig. 2*B*, bottom panel), consistent with the known contralateral visual field bias in these areas for saccadotopy stimuli.

**Figure 3.**
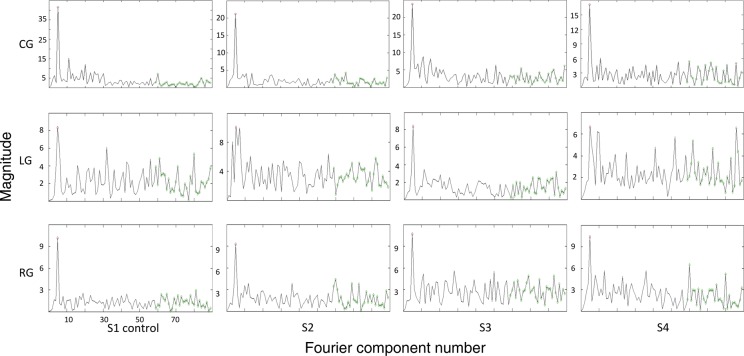
FS for each subject as a function of gaze condition. The cycles per run was “5” in the present study. As can be seen in each of each of the single subject left-hemispheric Fourier plots, there is a peak at “5” for the center-gaze condition. In contrast, the LG and RG (middle and bottom panels, respectively) showed a relatively lower magnitude peak at 5, and there were additional medium-to-high frequency cycles in the FS with the eyes deviated only slightly in the orbits.

We then investigated the overall quality of the data by calculating Fourier spectra (FS) of our functional MRI data within the PPC. The spectra can be used to estimate the contrast-to-noise ratio (CNR). Figure 3 shows the left-hemisphere FS for each subject for center, left, and right gaze across an entire session. (The same pattern was observed in the right hemisphere.) Importantly, the magnitude peaked at the periodicity of the stimulus in all gaze conditions; in our case, at the stimulus cycle of “5.” The CNR was substantially reduced for the left and right gaze positions relative to center gaze suggesting some less consistent phase representation in many of our voxels with the gaze deviated.

**Figure 4.**
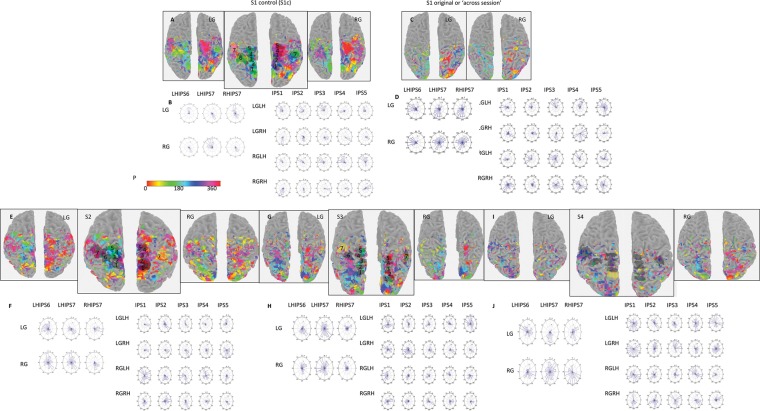
Phase maps and rose plots for IPS1 through IPS7 in the PPC for each subject. A comparison of surface renderings for the left (LG) and right (RG) starting gaze conditions. (*A*) A comparison for the complete within the session control subject (S1 control). The enlarged center panels (or “ROI definition” plots) represent LG but with the ROI drawn on the actual surfaces using the mrTools software for visualization of their relative locations in the inferior (IPL) and superior (SPL) parietal lobe. (*B*) The subsequent rose histograms (refer to the panels below) are based on these same ROIs. For both the phase and rose plots, 0° and 360° represent upward saccades relative to center, 180° represents downward saccades, 90° represents rightward saccades, and 270° represents leftward saccades. (*C*–*J*) S1 through S4 across-session data with the same conventions as S1c (above). S4 is presented without any labeling, for clear visualization of the ROI locations.

Figure 4 shows the phase maps and the corresponding rose histograms for every subject as a function of gaze position and hemisphere. The corresponding coherence maps for these phase maps were thresholded at *c* > 0.15. The pattern of results for S1 was the same for all the gaze conditions within a session or across sessions, which confirms the validity of separate session runs used in the other subjects. For the gaze-center condition, there was a strong contralateral bias for memory-guided saccades. It is important to emphasize that IPS6 and IPS7 show opposite phase relationships. IPS6, like other PPC subregions, represents contralateral space, whereas IPS7 represents ipsilateral space at gaze center. The other PPC subregions in the left hemisphere show consistent rightward memory-guided saccade (green/turquoise pixels), and the PPC subregions in the right hemisphere show consistent leftward memory-guided saccade (red/violet pixels) activity. However, although the same overall pattern was observed, the SNR was enhanced for S1 control (same session) when compared with S1 original (across sessions). The IPS1–IPS7 ROIs are indicated in the enlarged center panel for LG with corresponding ROI numerical values. These ROIs are displayed to indicate their relative locations on the actual surface maps and were drawn and displayed on these same surfaces using the mrTools software (refer to Materials and Methods).

**Figure 5.**
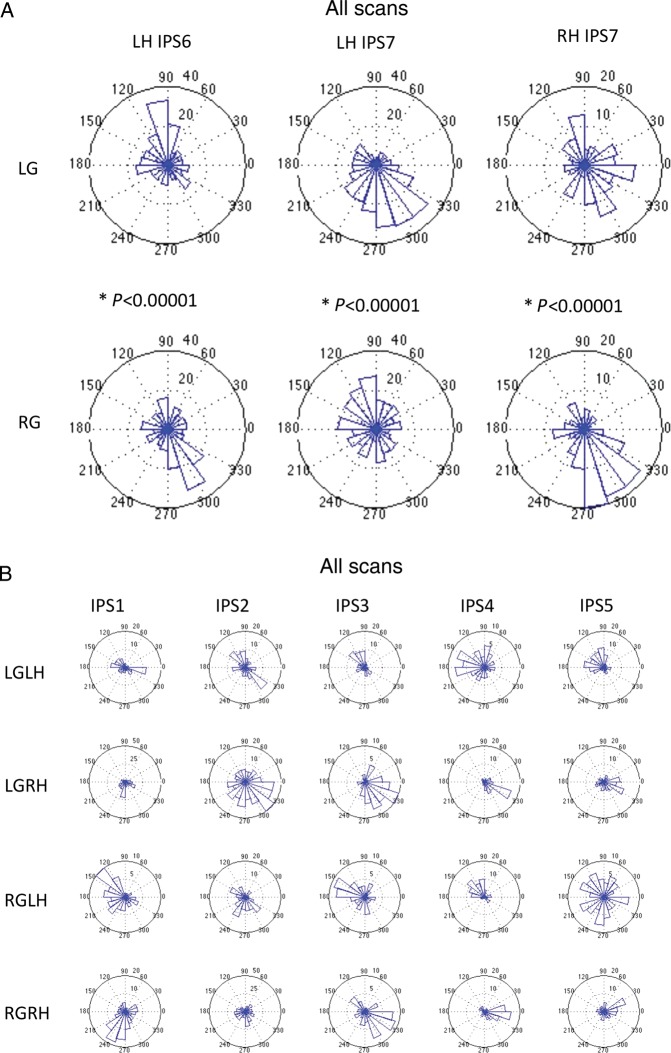
Rose histograms for IPS1 through IPS7 in the PPC pooled across all subjects. (*A*) Rose histograms for inferior PPC subregions (IPS6 and IPS7). 0° and 360° represent upward saccades relative to center, 180° represents downward saccades, 90° represents rightward saccades, and 270° represents leftward saccades. (*B*) Rose histograms for superior PPC subregions (IPS1–IPS5). ‘All scans’ refers to S1c to S4. S1 ‘across sessions’ was not included.

However, there are important differences in the distribution of phase values when the gaze was deviated from the head-/body midline (LG vs. RG conditions). As can be seen in the rose histograms for each subject, there are significant differences (*P* < 0.05 or <0.01) in the topographic representation of memory-guided saccadic endpoints for LG and RG shifts within the same hemisphere. For example, there was almost a 180° inversion of preferred phases for LG when compared with RG within the same hemisphere for IPS6 and IPS7 (Table 1, individual statistics). The same finding was also found when phase values were pooled across subjects, as shown in Figure 5. Overall, these findings provide evidence that several PPC subregions code in higher-order (head-/body-centered) coordinates.

As noted, eye tracking was conducted outside of the scanner in 4 naïve subjects using the identical stimuli, that is, with the RG and LG offset by ±4.1° when compared with CG. None of the statistical measures based on movement amplitude (main effects and interactions) were significant. Of prime importance, the movement amplitudes across the 3 different starting gaze positions using a repeated-measures analysis of variance were not significantly different (*F*_2,6_ = 0.252, *P* = 0.561). Therefore, our fMRI differences were not a result of variance in saccade amplitudes as a function of starting gaze position.

## Discussion

The present data demonstrate that the PPC not only encodes saccade direction in topographic maps, but also takes into account the position of the eyes in the orbits and reorganizes these maps accordingly. In particular, the topographic representation of visual space within a hemisphere differed depending on whether observers shifted their gaze slightly to the left or right of a central point aligned to the midline of the head and body. Our results suggest that there are many neurons throughout the PPC that encode visual- and saccade-related spatial information in higher-order reference frames, such as a head-/body-centered coordinate system, and these neurons appear to be clustered, such that the pattern is detectable using voxel-based fMRI. These effects were most pronounced in areas IPS6 and IPS7, but voxels that showed substantial changes in preference were also found in IPS2 (RH), IPS3 (LH), and IPS5 (LH). To our knowledge, this is the first evidence for higher-order coordinate system beyond an eye- or retina-centered coordinate system across the extent of the PPC in humans.

Two human fMRI studies have reported retino-centric coordinate mapping within the PPC (Fernandez-Ruiz et al. 2007; Van Pelt et al. 2010). In one of these studies, subjects wore displacing prisms while movements were executed from the midline. It was reported that neuronal responses in the PPC coded for the visual target even after it was displaced by the prisms, while they did not for the motor movement. However, scanning with prisms was performed only after adaptation to the prisms had occurred, and it may be the case that, under those circumstances, the PPC shows a form of plasticity that then conceals evidence of reorganization that may have been otherwise present. The second study examined repetition suppression and reported a “null effect” for the PPC (Van Pelt et al. 2010). Repetition suppression may not be sensitive enough to identify higher nonretinotopic reference frames with fMRI-blood oxygen level-dependent.

Other studies have reported evidence for eye-centered coding within the human PPC (DeSouza et al. 2000; Medendorp et al. 2003). These studies were designed to distinguish between retinal- and eye-centric reference frames, not between retinal/eye-centric and higher-order frames, such as head-/body-centered. If the PPC codes visual information in retinal/eye-centered reference frames, then the visual or saccade maps should not be affected by the position of the eye in the orbit. Thus, asking subjects to make identical memory-guided saccades from different starting positions should yield the same saccadotopic maps within the PPC. It could be the case that the maps abandon their strong contralateral preference and become highly distributed when the eye is deviated from center because of the existence of gain fields with many different orientations (Andersen 1997), all opposing one another, making the overall representation that can be derived by means of fMRI very noisy. Our analyses (Figs 4 and 5) suggest that this was not the case.

It has been argued that neurons in most of the PPC areas encode information in so-called “intermediate reference frames” (Duhamel et al. 1997; Mullette-Gillman et al. 2005, 2009), whereby different neurons within an area code in different (e.g., retinal or head-centered) or mixed reference frames. If this is true then we may expect some IPS areas to show small or nonsignificant differences in topographic representation as a function of the gaze position. This is what was found mostly for areas IPS1 and IPS4 where the distributions of phase shift angles were relatively narrowly centered around 0°. However, many of the subregions we identified showed different topographic representations for shifted gaze positions (relative to a central to the midline of the head and body), suggesting that a considerable number of neurons in the PPC encodes reference frames in higher-order coordinate systems. The subtle effects seen in our distributions of phase shifts in IPS1 and IPS4 may be due to the relatively small displacements of gaze direction used. It may be the case, that with larger shifts of the starting eye position we would find more voxels that show sizeable phase shifts because, for larger deviations of starting eye position, more saccade targets would shift from contralateral to ipsilateral relative to the head-/body-centered vertical meridian. An 8.2° shift in starting eye position would have shifted even the 3 and 9 o'clock targets across. Unfortunately, subjects' total visual FOV in the scanner did not allow for such large gaze shifts relative to center, as the most peripheral saccade targets would not have been visible on the screen inside the scanner. Future experiments will be necessary to resolve this question. It will be equally important to determine whether or not the voxels that we identified as “shifting” are more compatible with a head- or body-centered reference frame.

Two recent human fMRI studies reported a higher-order reference frame for a part of the PPC, and these results are particular pertinent to the present results (Sereno and Huang 2006; Pertzov et al. 2011). Our current results show that head-/body-centered coding is much more distributed in the PPC, that is, throughout subregions of the inferior (IPS6 and IPS7) and superior PPC (IPS2, IPS3, and IPS5). While our findings are the first to demonstrate such higher-order reference frames in large parts of PPC in the human, it is not entirely unexpected given the knowledge gained from single-unit recordings in monkey PPC and changes in neuronal responses during spatial updating. Neurons in the lateral intraparietal area show re-mapping of visual receptive fields before saccadic eye movements (Duhamel et al. 1992). Neurons in the medial superior temporal area show evidence for head-centered coding of heading direction (Bradley et al. 1996), and those in VIP can have receptive fields that are strictly head-/world-centered (Duhamel et al. 1997). In other words, the PPC has neurons that represent the transformation of visual information into a reference frame that allows for the calculation of motor error, and it is a relatively “downstream” output source.

The present results support one of the earliest but still controversial interpretations of the function of the PPC based on electrophysiological data (Mountcastle et al. 1975): PPC neurons participate in transformations from retinal to other coordinate frames. We found a number of areas that exhibit a reorganization of the representation of space in PPC with subtle changes in eye position. These dynamic reorganizations are incompatible with a strictly retinal/eye-centered coding scheme, but suggest that large clusters of neurons, which code in head-/body-centered reference frames, are distributed throughout the PPC. Future studies based on the approach used here should be able to determine whether such coding is mostly in head- or body-centered coordinates.

## Funding

Funding to pay the Open Access publication charges for this article was provided by the Wellcome Trust.
